# The Impact of the West Africa Ebola Outbreak on Obstetric Health Care in Sierra Leone

**DOI:** 10.1371/journal.pone.0150080

**Published:** 2016-02-24

**Authors:** Kim J. Brolin Ribacke, Alex J. van Duinen, Helena Nordenstedt, Jonas Höijer, Ragnhild Molnes, Torunn Wigum Froseth, AP Koroma, Elisabeth Darj, Håkon Angel Bolkan, AnnaMia Ekström

**Affiliations:** 1 Department of Public Health Sciences, Karolinska Institutet, Stockholm, Sweden; 2 Department of Surgery, St Olav Hospital, Trondheim, Norway; 3 CapaCare, Trondheim, Norway; 4 Unit of Biostatistics, Institute of Environmental Medicine, Karolinska Institutet, Stockholm, Sweden; 5 Department of Public Health and General Practice, Norwegian University of Science and Technology, Trondheim, Norway; 6 Ministry of Health and Sanitation, Freetown, Sierra Leone; 7 Department of Obstetrics and Gynaecology, St Olav Hospital, Trondheim, Norway; 8 Department of Cancer Research and Molecular Medicine, Norwegian University of Science and Technology, Trondheim, Norway; 9 Department of Infectious Diseases, Karolinska University Hospital, Stockholm, Sweden; King Abdullah International Medical Research Center, SAUDI ARABIA

## Abstract

**Background:**

As Sierra Leone celebrates the end of the Ebola Virus Disease (EVD) outbreak, we can begin to fully grasp its impact on already weak health systems. The EVD outbreak in West Africa forced many hospitals to close down or reduce their activity, either to prevent nosocomial transmission or because of staff shortages. The aim of this study is to assess the potential impact of EVD on nationwide access to obstetric care in Sierra Leone.

**Methods and Findings:**

Community health officers collected weekly data between January 2014—May 2015 on in-hospital deliveries and caesarean sections (C-sections) from all open facilities (public, private for-profit and private non-profit sectors) offering emergency obstetrics in Sierra Leone. This was compared to official data of EVD cases per district. Logistic and Poisson regression analyses were used to compute risk and rate estimates. Nationwide, the number of in-hospital deliveries and C-sections decreased by over 20% during the EVD outbreak. The decline occurred early on in the EVD outbreak and was mainly attributable to the closing of private not-for-profit hospitals rather than government facilities. Due to difficulties in collecting data in the midst of an epidemic, limitations of this study include some missing data points.

**Conclusions:**

Both the number of in-hospital deliveries and C-sections substantially declined shortly after the onset of the EVD outbreak. Since access to emergency obstetric care, like C-sections, is associated with decreased maternal mortality, many women are likely to have died due to the reduced access to appropriate care during childbirth. Future research on indirect health effects of health system breakdown should ideally be nationwide and continue also into the recovery phase. It is also important to understand the mechanisms behind the deterioration so that important health services can be reestablished.

## Introduction

On November 7^th^ 2015, the World Health Organization (WHO) finally declared Sierra Leone free of Ebola Virus Disease (EVD), but its consequences on an already fragile health system and overall morbidity and mortality are only starting to emerge. Since the first case of EVD was recorded in the Kailahun district in the Eastern province of Sierra Leone on May 24^th^ 2014, a total number of 8,704 people have been infected and 3,589 have died [1].

During the peak of the epidemic in October to December 2014, hospitals and health staff were overburdened with patients and health facilities were also potential hubs of transmission, given the severe shortage of proper equipment and lack of isolation capacity. Many health facilities had to close down or substantially reduce their activities when both staff and patients stopped coming, both in fear of infection or because patients were turned away or transferred to other facilities [2,3].

Even before the current EVD outbreak, Sierra Leone had one of the highest maternal mortality ratios and infant mortality rates in the world, with an adjusted maternal mortality ratio of 1100 maternal deaths per 100 000 live births [4]. This is despite an ambitious government program, in place since April 2010, which offers free healthcare for pregnant women, lactating mothers and children below five years [5]. Sierra Leone has a diverse health service delivery system with both private and public actors offering emergency obstetrics. In 2012, around 60% of all major surgery was performed by the private sector [2]. A health workforce assessment in 2008 found only seven obstetricians serving the whole population of Sierra Leone, leaving many women to seek help from traditional birth attendants [6,7]. At tertiary facilities (those included in this study), deliveries are often referrals from other health facility units; due to the potential need of emergency obstetric care. Before the EVD outbreak in Sierra Leone, approximately 50% of deliveries took place in any kind of health facility [8,9]. In 2012, the caesarean section (C-section) ratio in Sierra Leone ranged between 2.3% and 4.5% [8,9], which is below current WHO recommendations, suggesting a 10% population level of C-sections to prevent excess maternal mortality [10,11].

It is currently not known how the EVD outbreak has affected routine maternal care, including emergency obstetric care, but during the initial stages of the EVD outbreak in Sierra Leone (May-October 2014) a significant decline in general admissions and major surgery was observed and initial reports indicated substantial excess mortality rates [12–14]. To date, there are only a few studies trying to determine the impact of the EVD outbreak on maternal health published [15–17]. Therefore, this study quantifies the impact of EVD on obstetric health care, measured as C-sections and in-hospital deliveries at tertiary facilities, in Sierra Leone during the outbreak. We use data from all open health facilities offering emergency obstetrics within the country and compare the pre-outbreak period with both the outbreak peak period as well as the subsequent outbreak slow-down period when the number of cases rapidly decreased.

## Methods

This study was conducted as part of an on-going collaboration between the Ministry of Health and Sanitation (MoHS) in Sierra Leone, Karolinska Institutet in Sweden, the Norwegian University of Science and Technology, and the non-governmental organization CapaCare, and constitutes part of a new surveillance initiative to monitor the effects of the Ebola epidemic on health services [12]. The director of Research and Non-Communicable Diseases and the Director of Hospitals and Laboratory Services of the MoHS approved the study. Since data was collected retrospectively from operation theatre-, delivery-, and admission logbooks, patient consent was not possible to obtain. Therefore, all information concerning individual patients was anonymized and de-identified prior to analysis.

### Data collection

A countrywide study in 2013 systematically mapped all 61 governmental, private, not- and for-profit healthcare facilities that offer in-patient care and major surgery [2]. For our study, all these facilities were surveyed since September 2014, by 21 Community Health Officers (CHOs) (details described previously [12]). The CHOs were on leave from a surgical task sharing training program, due to restrictions on clinical training during the EVD outbreak. To minimize travel and potential exposure to EVD, most data collectors lived nearby, or had recently worked or practised in the facilities they regularly visited. During the first facility visit in the end of September 2014, retrospective data was retrieved for the first 38 weeks of the year, and thereafter on a bi-weekly basis until end of May 2015. Weekly accumulated numbers on deliveries and C-sections was collected from readily available operation theatre-, and admission logbooks. The data collectors were trained for one full day and coordinated locally by a final year medical student, also on leave due to the EVD epidemic. Data was captured on tablets (Huawei Mediapad 7 with data SIM connectivity) using the District Health Information System 2 (DHIS 2) software, designed for collection, validation and analysis of aggregated health data. Data was transferred via a secured Internet connection to a central cloud server and monthly validated.

Of visited facilities, 32 performed at least 5 deliveries and/or C-sections during the study period (week 1, 2014 to Week 20, 2015) and were included in our study. When this criterion was met, facilities did not need to be open consistently during all three periods, in fact, many of the included facilities closed after the onset of the EVD outbreak. A list of all hospitals included in this study can be found in S1 Appendix. Data on facility status as well as weekly numbers of deliveries and C-sections can be found in S2 Appendix.

Data on weekly number of EVD cases per district was retrieved from the MoHS in Sierra Leone and the WHO [18,19]. Since the first EVD case in Sierra Leone was reported in late May 2014, we define the pre-outbreak period as week 1 to 21, 2014 (period 1) and divide the post-outbreak into two separate periods; week 22 to 52, 2014 (period 2; outbreak peak) and week 1 to 20, 2015 (period 3; outbreak slow-down).

To understand expected seasonal variations in C-sections, we compared our 2014 and 2015 numbers with those from 2012. Data on the number of C-sections was collected retrospectively by 12 local medical students in 2013 from operation, anaesthesia, and delivery logbooks and used as a reference for these comparisons [2]. We were not able to obtain 2012 data on the number of in-hospitals deliveries.

### Data analysis

In order to determine how the number of in-hospital deliveries and C-sections varied over time, we calculated the mean weekly number of deliveries and C-sections over the three time periods. The mean weekly incidence rate ratios were calculated with corresponding 95% confidence intervals (CI), using Poisson regression with the pre-outbreak period as the reference. These numbers are calculated for the whole nation, by province, and by type of facility. EVD incidence rate per 100 000 inhabitants was calculated using population data [8]. Logistic regression was used to compute 95% CI around the C-section proportions. The association between the number of deliveries and C-sections and the number of EVD cases was estimated using Poisson regression, treating the number of EVD cases as the predictor variable. The estimates are incidence rate ratios (IRR) for an increase of 100 EVD cases.

To compensate for some missing observations on deliveries and C-sections, we used two different imputation methods: one where a missing value was interpreted as a zero, and one where the last observed value for the specific facility was used. The first method is the one presented in the paper. However, the difference between the two imputation methods was negligible, indicating that missing values did not influence the results in any significant way.

## Results

### Ebola Virus Disease in Sierra Leone

**Fig 1:**
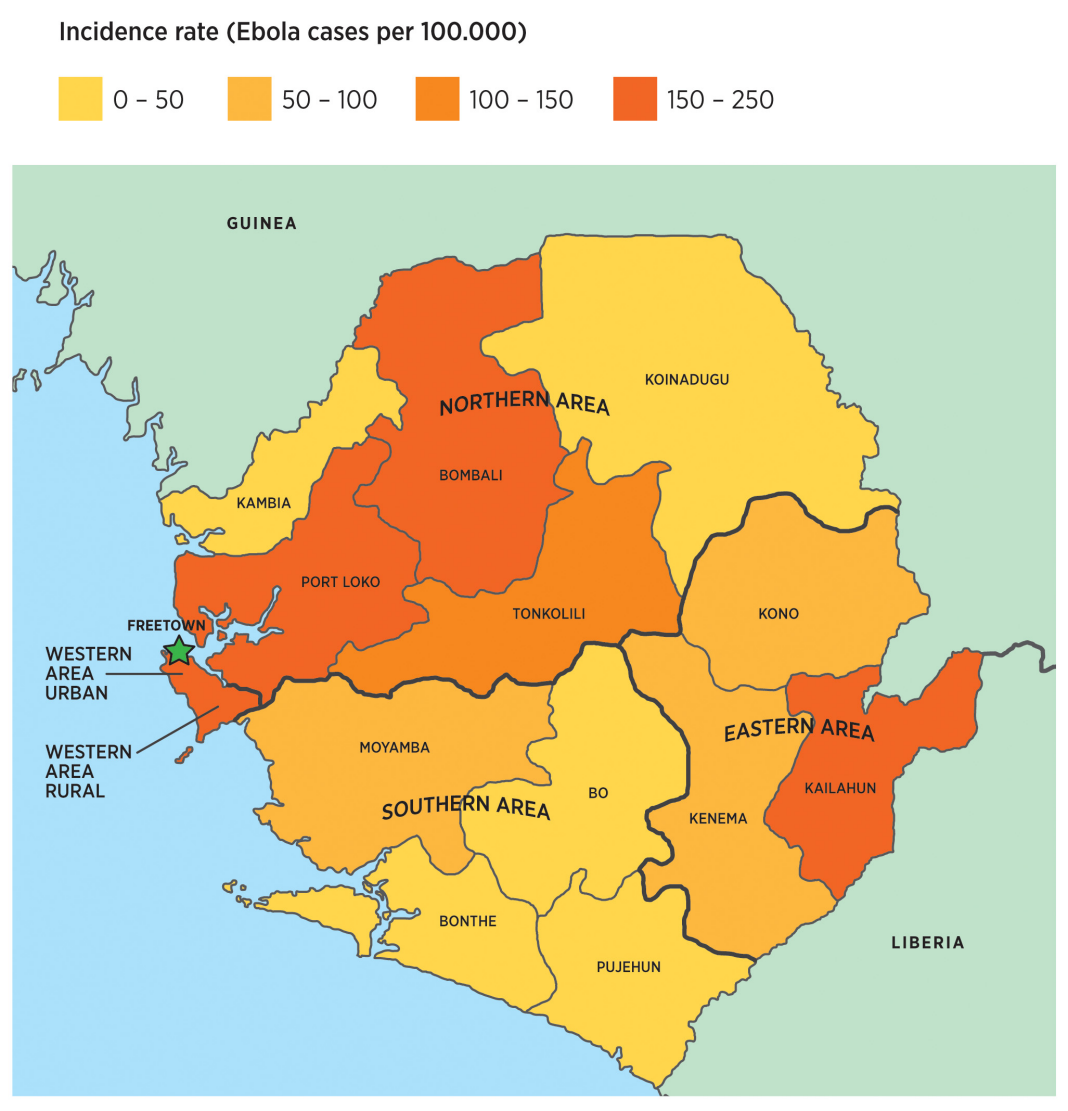
Burden of EVD in Sierra Leone EVD incidence rate per 100 000 inhabitants shown per district and province.

Following the first EVD case in May 2014 in Kailahun district, Eastern province, the spread of the virus continued into the remaining districts in this province and then onwards to the Southern and Northern provinces, reaching the Western Area by August 2014. The pattern was similar with the rapid increase of cases, peaking in August 2014 in the Eastern province, and in November 2014 in both the Northern, and Western provinces. All provinces showed a decline, which began in January 2015. To estimate the burden of EVD per district and province, we calculated the EVD incidence rate per 100 000 inhabitants for the whole study period (Fig 1) [8, 18,19].

### In-hospital deliveries and C-sections before and during the EVD outbreak

**Fig 2:**
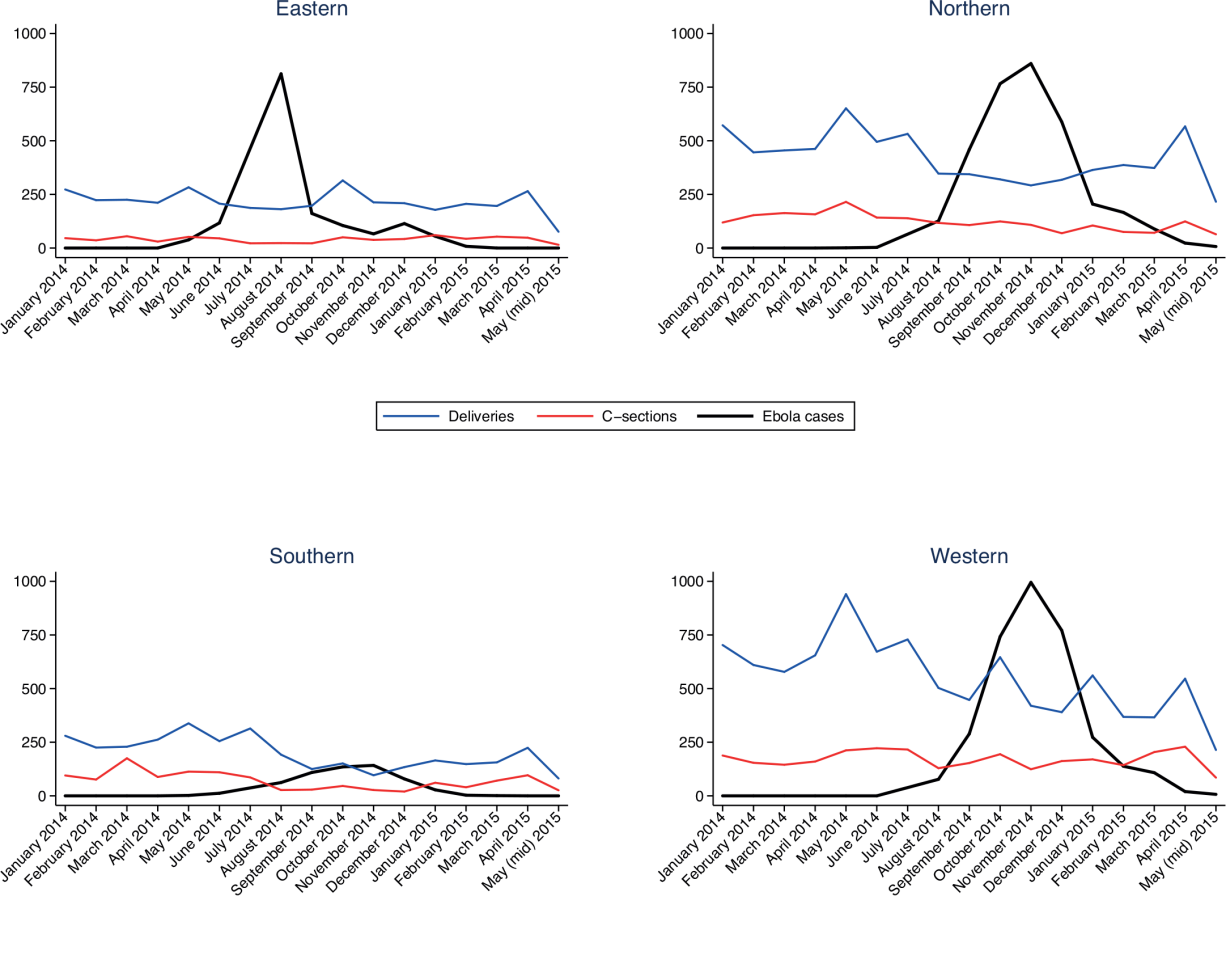
In-hospital deliveries and C-sections in relation to the simultaneous burden of EVD. Monthly data on number of in-hospital deliveries (blue), C-sections (red) and new EVD cases (black), shown per province.

Nationwide, the number of in-hospital deliveries and C-sections decreased by over 20% in both periods 2 and 3 compared to period 1, albeit with substantial variation between districts and provinces (Table 1). The decline in number of in-hospital deliveries ranged from less than 10% in the Eastern province, up to almost 40% in the Southern province and Western Area (Table 1). In the Eastern province, which was the first hit by the EVD outbreak, the number of C-sections decreased by 20% in period 2 but then increased by 10% in period 3, compared to the pre-outbreak period. In the Western area, there was also a slight increase in the number of C-sections performed in period 2 and period 3 compared to period 1. In both the Northern and Southern provinces, the number of C-sections decreased by almost 20% and over 50% respectively in period 2, and by over 40% in period 3 for both provinces. The changes in volume of in-hospital deliveries and C-sections (absolute numbers) in relation to the simultaneous burden of EVD (as measured by new confirmed EVD cases in absolute numbers, per province and month) are visualized in Fig 2 The decrease in number of C-sections was significantly associated with the number of EVD cases for all provinces, whereas in-hospital deliveries were associated with the number of EVD cases in all but the Eastern province (Table 1).

**Table 1:** In-hospital deliveries and C-sections in Sierra Leone and association of EVD case load and decrease in in-hospital deliveries and C-sections. N = 32.

	Period 1 (pre-outbreak)	Period 2	Period 3	EVD case load and decrease in obstetric care			
Deliveries	Mean	Mean (% change)	Mean (% change)	IRR	p-value	95% CI	
**Nationwide**	394	312 (-21%)*	283 (-28%)*				
**Eastern**	55	51 (-7%)*	46 (-16%)*	0.984	0.089	0.966	1.002
**Northern**	117	90 (-23%)*	95 (-19%)*	0.936	0.000	0.927	0.945
**Southern**	62	42 (-32%)*	39 (-37%)*	0.618	0.000	0.570	0.671
**Western**	159	130 (-18%)*	103 (-35%)*	0.967	0.000	0.960	0.974
**C-sections**							
**Nationwide**	112	88 (-22%)*	89 (-20%)*				
**Eastern**	10	8 (-20%)*	11 (10%)	0.926	0.004	0.880	0.975
**Northern**	37	27 (-17%)*	22 (-41%)*	0.955	0.000	0.939	0.971
**Southern**	26	12 (-54%)*	15 (-42%)*	0.382	0.000	0.325	0.449
**Western**	39	40 (3%)	42 (8%)	0.982	0.004	0.970	0.994

Table 1 shows in-hospital deliveries and C-sections in Sierra Leone nationwide and per province for period 1 (baseline), 2 and 3. * = significant change (Poisson regression). Also shown is the association of number of EVD cases and decrease in in-hospital deliveries and C-sections, per province.

**Fig 3:**
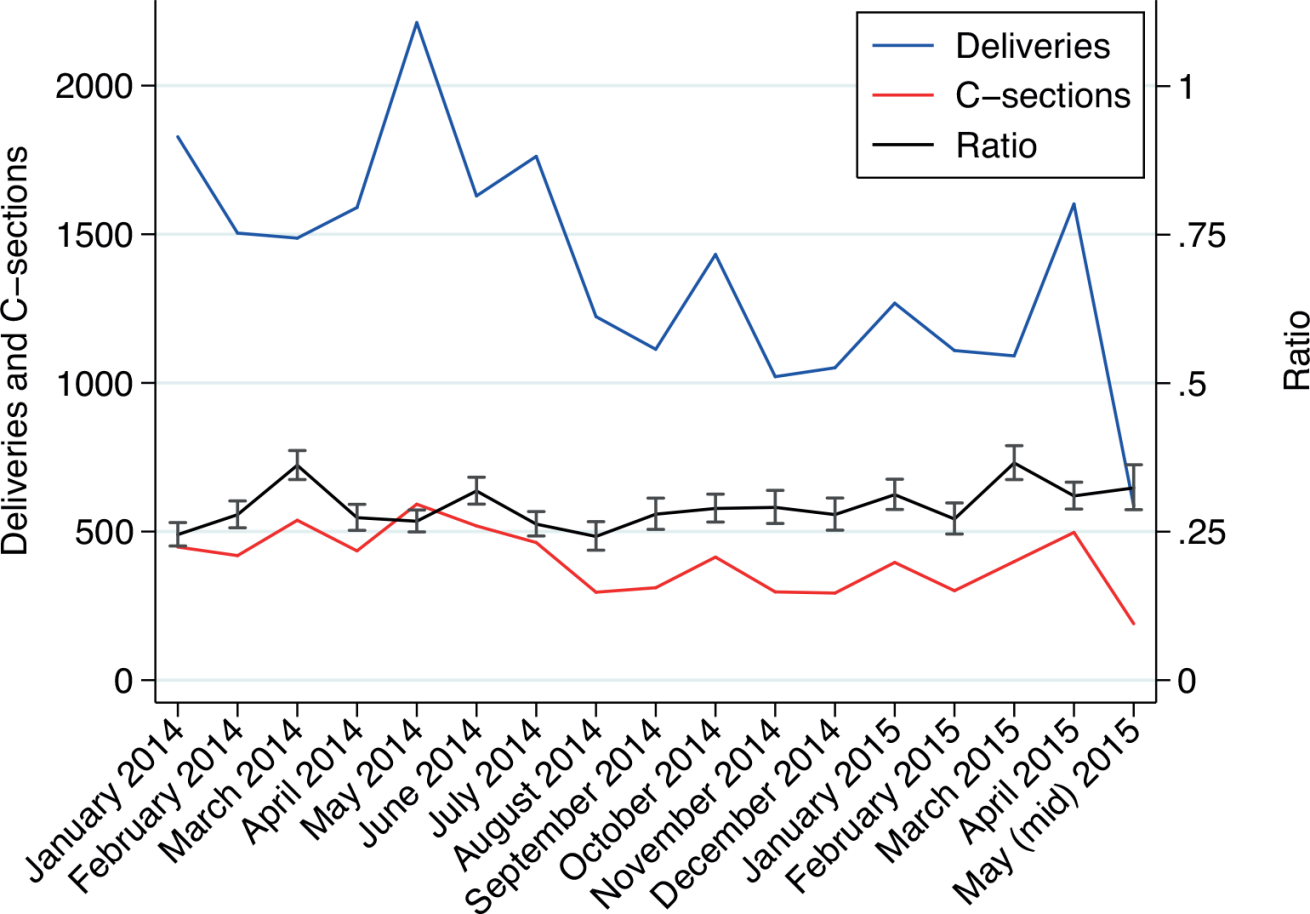
Monthly number of in-hospital deliveries and C-sections nationwide during the study period. Also shown is the ratio of C-sections of all in-hospital deliveries with a 95% CI.

Fig 3 shows nationwide changes in absolute numbers of in-hospital deliveries (blue line) and C-sections (red line) per month during 2014, as well as the ratio between the number of C-sections and the total number of deliveries (calculated as the proportion of C-sections out of total number of deliveries) with 95% CI. The ratio between C-sections and deliveries did not change substantially overall, but as is described below, there was a clear difference by type of health care sector.

**Fig 4:**
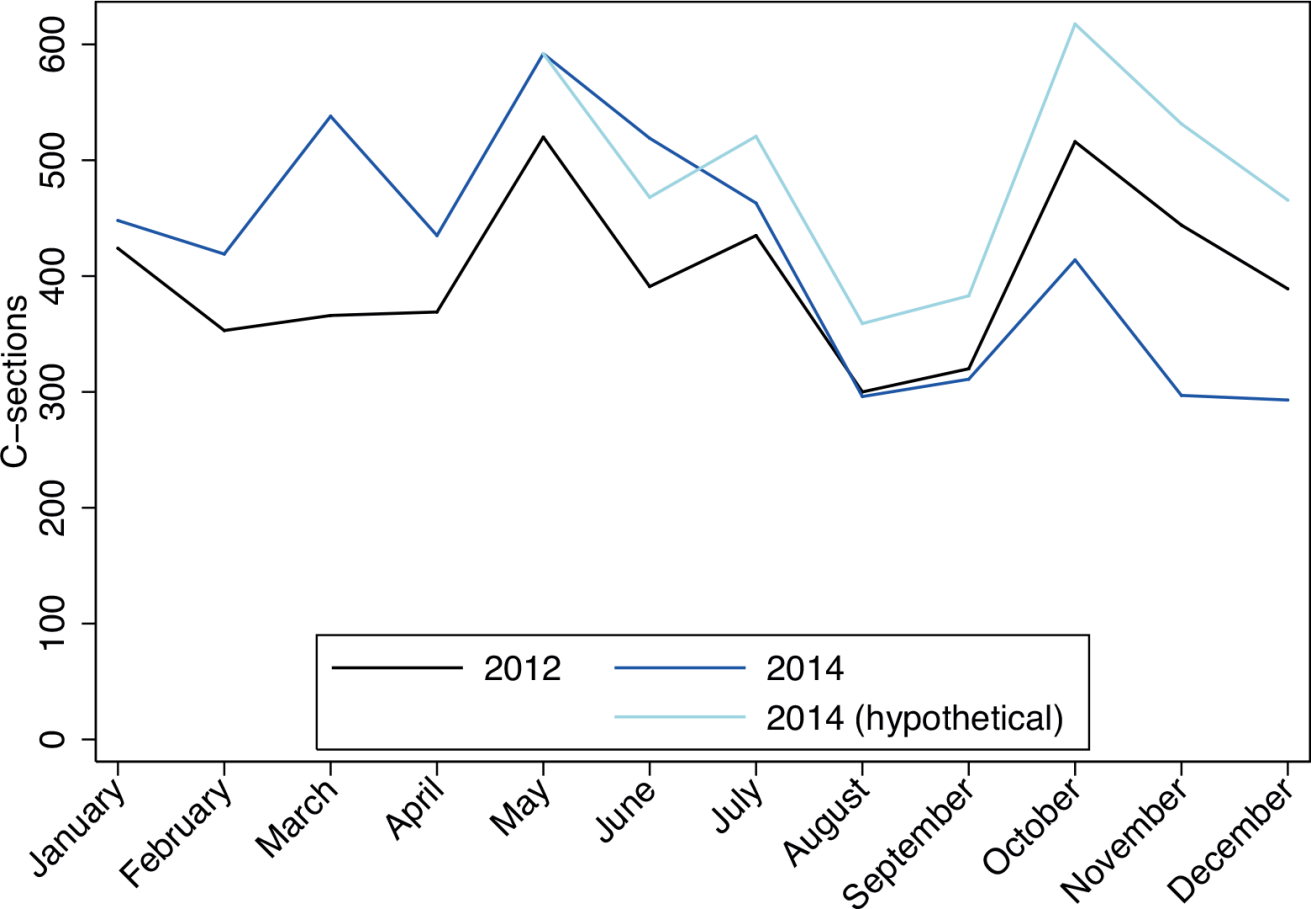
Seasonal variations of number of C-sections in 2012 (black), 2014 (dark blue) and hypothetical numbers (light blue).

We found seasonal variations in terms of C-sections, both in 2012 and in 2014, when heavy rainfalls in May to November cause a decline in the volume. Fig 4 shows the number of C-sections per month nationwide in 2012 (black) and 2014 (bright blue). In absolute numbers, 4827 C-sections were performed in 2012 [2] versus 5025 in 2014, corresponding to a 4.1% increase. However, this whole increase was due to a higher C-section volume in the first part of 2014. We can speculate that in the absence of EVD, the improvement seen in the first part of 2014 would have continued at a similar level for the remaining weeks. In order to provide an estimate of hypothetical number of C-sections after the onset of the EVD outbreak in 2014, we calculated the proportional increase in the pre-outbreak period and extrapolated this onto the remaining months in 2014 (light blue).

### Performance per type of health care sector

The majority of all health facilities in Sierra Leone included in this study are run by the government (n = 18 [58%]), or the private non-profit sector (n = 13 [42%]). Only one of the providers was private for-profit and accounted for less than 1% of both deliveries and C-sections in 2014. The number of health facilities performing in-hospitals deliveries and C-sections per health care provider sector in Sierra Leone in 2014 is shown in Table 2. The governmental facilities accounted for 76% of in-hospital deliveries and 67% of C-sections recorded in 2014. The private non-profit hospitals performed 24% of the total number of in-hospital deliveries and 33% of the C-sections volume. After the onset of the EVD outbreak, the government facilities reduced their volume of deliveries by 15% in period 2 and 36% in period 3. However, the number of C-sections only decreased by 5% in period 2 and actually increased slightly by 5% in period 3. Within private non-profit hospitals, the corresponding decrease in in-hospital deliveries amounted to 37% and 5% in periods 2 and 3, respectively. The number of C-sections performed by these types of facilities decreased by around 50% in both post-Ebola periods (Table 3). Due to the low number of deliveries and C-sections done at private for-profit facilities, we did not calculate the percent change.

**Table 2:** 2014 Performance per type of health care sector

2014		Nr of facilities	Deliveries	C-sections	% of all deliveries*	% of all C-sections*
	**Governmental**	18	13545	3344	76%	67%
	**Private non-profit**	13	4257	1649	24%	33%
	**Private for- profit**	1	40	32	<1%	<1%
	**Total**	32	17842	5025	100%	100%

*Round off to closest whole number

**Table 3:** In-hospital deliveries and C-sections in Sierra Leone

	Period 1 (pre-outbreak)	Period 2	Period 3
Deliveries	Mean	Mean (% change)	Mean (% change)
**Governmental**	288	244 (-15%)	183 (-36%)
**Private non-profit**	105	67 (-37%)	100 (-5%)
**C-sections**			
**Governmental**	67	63 (-5%)	70 (5%)
**Private non-profit**	45	23 (-49%)	19 (-58%%)

Table 3 shows numbers nationwide and per health care provider sector for period 1, 2 and 3 where period 1 serves as baseline. Weekly mean in absolute numbers as well as % change is shown for both in-hospital deliveries and C-sections.

## Discussion

This unique study is built on data collected in the midst of the devastating EVD outbreak in Sierra Leone. It includes weekly information from all facilities performing C-sections during 2014 and the first 20 weeks of 2015, including both governmental and private facilities, which enables us to analyse how different types of facility ownership were affected by the outbreak. In this study, we found a nationwide decrease in the number of in-hospital deliveries and C-sections by over 20% during the EVD outbreak. Most of this decline occurred early in the EVD outbreak and was mainly attributable to the closing of private not-for-profit facilities rather than government facilities.

### Unmet Obstetric Need

The crude birth rate (live births per 1000 population) in Sierra Leone was 36 between 2010–2012, giving an estimated 223 200 babies born per year [8]. In 2014, 23 499 deliveries took place at the hospitals included in this study and 5025 C-sections were performed over the same period. Using the WHO recommendations of a 10% C-section ratio [10,11], the annual number of C-sections performed in Sierra Leone should have been approximately 22 320 (10% of the 223 200 births per year). The actual number of 5025 C-sections in 2014 only amounts to a C-section ratio of less than 2.5%, or a fourth of what would be needed to avoid excessive maternal death, leaving roughly 17 000 women in need of a C-section being untreated. The impact of the EVD outbreak is clear, with a much lower C-section volume in the second half of 2014 as compared to the pre-outbreak period. The average weekly number of C-sections in period 1 was about 20% higher than in period 2 and 3 (Table 1), meaning that even less C-sections took place after the onset of the EVD outbreak.

During the first weeks of the EVD outbreak, the volume of in-hospital deliveries and C-sections was similar to or above that of the baseline period (Fig 2). As the number of EVD cases increased over the subsequent weeks, a corresponding decline in obstetric hospital services was apparent across all provinces. The decline in in-hospital deliveries was most pronounced in the Southern province and Western area, whereas the decline in C-sections was highest in the Northern and Southern provinces. In all provinces, there is a significant association between the number of EVD cases and decrease in number of C-sections. In terms of in-hospital deliveries, EVD load is associated with a decrease in in-hospital deliveries in all provinces but the Eastern province, where this failed to be statistically significant (Table 1). While this study is focused on in-hospital deliveries and C-section volume, the decrease in deliveries suggest that also other emergency obstetric care signal functions such as assisted vaginal delivery and manual removal of the placenta decreased after the onset of the EVD outbreak.

### Ratio Deliveries /C-sections

Our analyses show that despite the EVD outbreak, most hospitals continued to perform the same percentage of C-sections in women reaching the hospital for delivery as in the pre-EVD period (i.e. the in-hospital C-sections ratio remains around 28%), albeit at lower absolute numbers. One possible explanation is that in Sierra Leone, deliveries taking place at tertiary facilities, such as those described in this study, are in general, high-risk deliveries, having been referred from a primary health care unit or similar. This explains the relatively high C-section ratio seen at the included hospitals and also the unchanged ratio between the number of deliveries and the number of C-sections during the EVD outbreak.

### In-hospital deliveries and C-sections by health care sector and governmental efforts

One of the most effective means of reducing maternal mortality is the provision of C-sections for all women who need them [20]. The increase in C-section volumes in the first half of 2014 was probably an effect of the efforts of the Sierra Leonean government to improve maternal health [5]. Almost the entire increase in the first half of 2014 could be attributed to an increased capacity in the governmental sector, further highlighting the increasingly important role of the governmental health care service sector in the recovery of the health system post-Ebola. In terms of in-hospital deliveries, the decrease was most apparent in the post outbreak period for governmental facilities but in peak outbreak for private non-profit facilities. It is apparent from Table 2 that the bulk of the decrease in C-sections in the second half of 2014 was due to decreased activity by private non-profit hospitals. We found that the governmental sector did not reduce their provision of C-sections much after the onset of the outbreak, whereas the C-sections volume by the private non-profit sector was almost halved. The large decrease in C-sections volume by the private non-profit sector pre- and post-Ebola can in part be explained by the closure of the Gondoma Referral Center run by MSF (Médecins sans Frontières) in Bo district, Southern province. Up until week 32, this hospital performed an average of 20 deliveries and 12 C-sections weekly in 2014 (S2 Appendix). Instead, MSF opened an Ebola Management Unit. Several other private non-profit facilities also shifted their focus area from ordinary medical services towards Ebola related services. In general, at hospital level, those facilities that remained open performed about the same number of deliveries and C-sections after the onset of the EVD outbreak, indicating that the decrease instead depends on the closing of key health facilities. This exacerbates the inequality in accessing emergency obstetric care, here measured as deliveries at tertiary facilities and C-sections, dependent on where in the country a woman lives, and highlights the need for equity in access to health services.

### Study strengths and limitations

Our study greatly benefits from being nationwide and encompassing both the period immediately before the outbreak as well as the slow-down period when the number of cases rapidly declined. Due to our strong network within Sierra Leone, we were able to rapidly initiate this study and continuously collect data during the EVD outbreak.

Data was retrieved from surgical records and medical logbooks, and there is a possibility that not all procedures were registered or registered correctly. Due to the difficulties in collecting data in the midst of an epidemic, some 2015 data was missing. We treated missing data as zero, further underestimating the magnitude of the unmet need for in-hospital deliveries and C-sections.

## Conclusions

Before the EVD outbreak, Sierra Leone had a positive trend in terms of access to obstetric care despite a remaining severe unmet need. However, the improvement seen in the first half of 2014 was abruptly halted by the EVD outbreak. We speculate that, if the EVD outbreak had not hit Sierra Leone, this positive trend would have continued. Thus, the significant dent in the number of pregnant women accessing and receiving tertiary skilled care observed in this study, probably underestimates the true unmet need for skilled obstetric care in Sierra Leone. The full magnitude of the damage attributable to the Ebola outbreak is yet to be seen. This study is the first to quantify the initial Ebola-related decrease in nationwide access to life-saving obstetric procedures measured as C-sections and in-hospital deliveries, and also considers the mechanisms behind this reduction. This shows the importance of reestablishing reproductive health services including tertiary care to reduce and prevent excess maternal and infant mortality.

## Supporting Information

S1 Appendix List of all included health facilities. (PDF)

S2 Appendix Dataset. (PDF)
