# Supplementary material for: The Impact of the West Africa Ebola Outbreak on Obstetric Health Care in Sierra Leone
Source: PLoS One. 2016 Feb 24;11(2):e0150080. doi: 10.1371/journal.pone.0150080 (PMC4766087; doi:10.1371/journal.pone.0150080)
Supplement: S1 Appendix — (PDF) [file pone.0150080.s001.pdf]

Name of facility	District	Province	Type of facillity	Owner
Aberdeen Woman Center	Western Area	Western Area (Urban plus rural)	District hospital	PNP
Bai Bureh Memorial Community Hospital	Port Loko	Northern	District hospital	PNP
Bo Governmental Hospital	Bo	Southern	Tertiary hospital	Gov
Bonthe Governmental Hospital	Bonthe	Southern	District hospital	Gov
Choitram Hospital, Freetown	Western Area	Western Area (Urban plus rural)	Tertiary hospital	PNP
Gondama Refferal Center, Bo	Bo	Southern	District hospital	PNP
Kabala Governmental Hospital	Koinadugu	Northern	District hospital	Gov
Kailahun Governmental Hospital	Kailahun	Eastern	District hospital	Gov
Kamakwie Wesleyan Hospital	Bombali	Northern	Provincial hospital	PNP
Kambia Governmental Hospital	Kambia	Northern	District hospital	Gov
Kenema Governmental Hospital	Kenema	Eastern	Tertiary hospital	Gov
KingHarman Rd. Hospital	Western Area	Western Area (Urban plus rural)	Provincial hospital	Gov
Koidu Governmental Hospital	Kono	Eastern	Provincial hospital	Gov
Lion Heart Medical Center	Tonkolili	Northern	Provincial hospital	PNP
Lumley Governmental Hospital	Western Area	Western Area (Urban plus rural)	Provincial hospital	Gov
Lungi Governmental Hospital, Port Loko	Port Loko	Northern	District hospital	Gov
Magbenteh Community Hospital	Bombali	Northern	Provincial hospital	PNP
Magburaka Governemtnal Hospital	Tonkolili	Northern	District hospital	Gov
Makeni Governmental Hospital	Bombali	Northern	Provincial hospital	Gov
Masanga Hospital	Tonkolili	Northern	District hospital	PNP
Moyamba Govermental Hospital	Moyamba	Southern	Provincial hospital	Gov
Nixon Memorial Segbwema	Kailahun	Eastern	District hospital	PNP
Panguma Mission Hospital	Kenema	Eastern	Provincial hospital	PNP
PCM Hospital, Freetown	Western Area	Western Area (Urban plus rural)	Tertiary hospital	Gov
Port Loko Govermental Hospital	Port Loko	Northern	District hospital	Gov
Pujehun Governmental Hospital	Pujehun	Southern	Provincial hospital	Gov
Ralpha Maternity Hospital (Dr. Samba)	Western Area	Western Area (Urban plus rural)	Clinic	PP
Serabu Catholic Hospital	Bo	Eastern	Provincial hospital	PNP
St. John of God Catholic Hospital, Lunsar	Port Loko	Northern	Provincial hospital	PNP
UMC Matru Hospital	Bonthe	Southern	District hospital	PNP
Waterloo CHC	Western Area	Western Area (Urban plus rural)	Clinic	Gov
Wilberforce Military Hospital	Western Area	Western Area (Urban plus rural)	Tertiary hospital	Gov
