# Supplementary material for: The Impact of the West Africa Ebola Outbreak on Obstetric Health Care in Sierra Leone
Source: PLoS One. 2016 Feb 24;11(2):e0150080. doi: 10.1371/journal.pone.0150080 (PMC4766087; doi:10.1371/journal.pone.0150080)
Supplement: S2 Appendix — (PDF) [file pone.0150080.s002.pdf]

		Week	1	2	3	4	5	6	7	8	9	10	11	12	13	14	15
Kailahun governmental Hospital	Facility status	0 = closed	1	1	1	1	1	1	1	1	1	1	1	1	1	1	1
	Deliveries		7	13	7	11	10	3	6	7	5	7	10	9	3	8	8
		C-Section	1	3	1	1	3	1	1	1	3	6	3	0	5	2	6
Kenema Governmental Hospital	Facility status	0 = closed	1	1	1	1	1	1	1	1	1	1	1	1	1	1	1
	Deliveries		28	25	29	32	28	36	34	42	24	42	34	33	34	32	34
		C-Section	9	2	2	2	2	1	4	3	3	8	1	0	0	0	0
Panguma Hospital	Facility status	0 = closed	1	1	1	1	1	1	1	1	1	1	1	1	1	1	1
	Deliveries		4	5	6	4	5	1	4	2	4	8	6	4	4	6	4
		C-Section	0	1	2	1	0	1	0	0	2	1	1	1	0	0	1
Koidu Governmental Hospital	Facility status	0 = closed	1	1	1	1	1	1	1	1	1	1	1	1	1	1	1
	Deliveries		9	10	13	11	13	9	15	7	14	18		3	2	7	1
		C-Section	0	1	7	7	0	0	3	5	6	7	8	6	5	2	0
Nixon Memorial Segbwema	Facility status	0 = closed	1	1	1	1	1	1	1	1	1	1	1	1	1	1	1
	Deliveries		0	1	0	1	1	3	2	3	2	2	2	2	2	4	0
		C-Section	0	1	0	0	0	1	0	1	0	0	3	0	0	0	1
Kamakwie Wesleyan Hospital	Facility status	0 = closed	1	1	1	1	1	1	1	1	1	1	1	1	1	1	1
	Deliveries		4	4	1	1	3	7	6	8	5	7	3	6	5	8	4
		C-Section	2	2	1	2	0	4	1	3	2	1	1	3	2	5	2
Magbenteh Community Hospital	Facility status	0 = closed	1	1	1	1	1	1	1	1	1	1	1	1	1	1	1
	Deliveries		10	2	2	2	5			6	4	5	1	4	2	3	3
		C-Section	3	0	0	0	2			2	2	0	4	3	0	0	3
Makeni Governmental Hospital	Facility status	0 = closed	1	1	1	1	1	1	1	1	1	1	1	1	1	1	1
	Deliveries		38	41	34	42	26	20	30	23	27	42	20	30	32	31	36
		C-Section	8	3	6	5	7	7	6	8	7	5	10	8	7	7	3
Kambia Governmental Hospital	Facility status	0 = closed	1	1	1	1	1	1	1	1	1	1	1	1	1	1	1
	Deliveries		2	11	13	15	14	6	11	12	20	7	10	9	14	15	11
		C-Section	0	3	0	4	3	3	7	5	4	2	4	3	4	1	4
Kabala Governmental Hospital	Facility status	0 = closed	1	1	1	1	1	1	1	1	1	1	1	1	1	1	1
	Deliveries		14	14	22	17	21	14	11	25	18	22	16	15	9	14	21
		C-Section	1	1	2	0	0	1	3	0	0	4	1	2	0	6	6
Bai Bureh Memorial Community Hospital	Facility status	0 = closed	1	1	1	1	1	1	1	1	1	1	1	1	1	1	1
	Deliveries		7	1	1	1	3	3	1	3	0	4	4	0	0	0	1
		C-Section	1	0	1	0	0	0	0	0	1	1	0	0	0	0	1
[illegible]	Facility status	0 = closed	1	1	1	1	1	1	1	1	1	1	1	1	1	1	1

Lung Govern enta Facilit Port Lc	Deliveries		0	7	3	0	9	8	6	17	4	4	4	3	6	7	7
		C-Section	0	2	1	0	3	1	2	3	2	0	1	1	4	2	0
Port Loko Governm ental Hospital	Facility status	0 = closed	1	1	1	1	1	1	1	1	1	1	1	1	1	1	1
	Deliveries		7	18	14	15	0	9	14	8	11	10	16	19	13	16	9
		C-Section	0	1	2	0	0	0	2	0	6	7	4	1	4	0	2
St. John of God Catholic Hospital	Facility status	0 = closed	1	1	1	1	1	1	1	1	1	1	1	1	1	1	1
	Deliveries		3	5	10	5	9	8	7	6	5	6	3	6	8	4	9
		C-Section	2	10	9	4	2	7	6	8	9	19	11	7	6	11	13
Lion Heart Medical Center	Facility status	0 = closed	1	1	1	1	1	1	1	1	1	1	1	1	1	1	1
	Deliveries		2	3	4	3	3	4	3	2	14	8	2	6	4	2	3
		C-Section	1	1	1	1	1	0	1	1	2	1	0	2	1	0	2
Magburak a Governm ental Hospital	Facility status	0 = closed	1	1	1	1	1	1	1	1	1	1	1	1	1	1	1
	Deliveries		14	16	5	8	4	15	9	5	2	17	12	15	8	11	16
		C-Section	1	5	2	1	7	9	4	7	9	4	8	6	6	8	3
Masanga Hospital	Facility status	0 = closed	1	1	1	1	1	1	1	1	1	1	1	1	1	1	1
	Deliveries		6	4	7	9	8	6	7	9	7	6	3	5	4	3	4
		C-Section	1	1	0	2	1	1	2	4	1	2	1	1	1	2	1
Bo Governm ental Hospital	Facility status	0 = closed	1	1	1	1	1	1	1	1	1	1	1	1	1	1	1
	Deliveries		10	12	19	7	6	9	20	19	16	5	7	20	9	19	15
		C-Section	0	1	4	1	3	2	3	16	8	0	0	4	0	0	0
Gondama Refferal Center (MSF Bo)	Facility status	0 = closed	1	1	1	1	1	1	1	1	1	1	1	1	1	1	1
	Deliveries		13	28	20	23	20	10	16	12	12	21	24	26	21	25	30
		C-Section	9	18	11	15	7	5	9	7	4	10	13	14	18	22	15
Serabu Catholic Hospital	Facility status	0 = closed	1	1	1	1	1	1	1	1	1	1	1	1	1	1	1
	Deliveries		6	4	10	2	6	7	6	16	4	5	3	2	6	2	6
		C-Section	4	3	2	3	0	2	0	2	3	2	0	50	38	0	3
Bonthe Governm ental Hospital	Facility status	0 = closed	1	1	1	1	1	1	1	1	1	1	1	1	1	1	1
	Deliveries		6	4	3	3	2	6	5	5	3	3	7	4	2	0	3
		C-Section	0	0	0	0	0	0	0	0	0	0	0	0	0	0	0
UMC Mattru Hospital	Facility status	0 = closed	1	1	1	1	1	1	1	1	1	1	1	1	1	1	1
	Deliveries		4	2	3	6	3	3	4	1	6	4	4	6	2	1	3
		C-Section	0	1	0	1	1	0	0	0	1	0	1	1	1	0	0
Moyamba Governm ental Hospital	Facility status	0 = closed	1	1	1	1	1	1	1	1	1	1	1	1	1	1	1
	Deliveries		2	8	10	4	6	6	2	6	7	5	6	10	9	12	4
		C-Section	0	0	1	3	1	0	0	3	1	1	1	2	1	2	0
un nm l tal	Facility status	0 = closed	1	1	1	1	1	1	1	1	1	1	1	1	1	1	1

Pujeh Govern enta Hospi	Deliveries		10	3	7	4	4	10	4	4	6	4	4	4	6	4	4
		C-Section	3	0	0	3	0	2	3	4	1	4	4	4	6	4	4
Aberdeen Woman Center	Facility status	0 = closed	1	1	1	1	1	1	1	1	1	1	1	1	1	1	1
	Deliveries		25	29	27	22	34	25	38	22	32	27	24	26	27	24	28
		C-Section	3	6	8	4	10	9	9	5	2	7	7	7	7	4	5
Choitram Hospital	Facility status	0 = closed	1	1	1	1	1	1	1	1	1	1	1	1	1	1	1
	Deliveries		4	14	12	16	17	13	10	14	12	13	10	16	14	14	16
		C-Section	2	3	6	4	4	4	4	3	3	5	0	4	2	4	3
King Harman Rd. Hospital	Facility status	0 = closed	1	1	1	1	1	1	1	1	1	1	1	1	1	1	1
	Deliveries		1	3	7	12	8	7	5	6	7	0	0	5	8	3	8
		C-Section	0	1	0	0	0	0	0	0	0	0	0	0	0	0	1
Dr Sambas clinic	Facility status	0 = closed	0	0	0	0	0	0	0	0	0	0	0	0	0	0	0
	Deliveries		0	0	0	0	0	0	0	0	0	0	0	0	0	0	0
		C-Section	0	0	0	0	0	0	0	0	0	0	0	0	0	0	0
Lumley Governm ental Hospital	Facility status	0 = closed	1	1	1	1	1	1	1	1	1	1	1	1	1	1	1
	Deliveries		0	9	10	5	8	13	10	10	12	18	11	10	0	11	14
		C-Section	0	1	0	1	0	0	0	0	0	0	0	0	0	2	0
Waterloo CHC	Hospital status	0 = closed	1	1	1	1	1	1	1	1	1	1	1	1	1	1	1
	Deliveries		13	19	24	23	24	26	22	26	24	15	19	19	25	32	28
		C-Section	0	0	0	0	0	0	0	0	0	0	0	0	0	0	0
Wilberfor ce Military Hospital	Hospital status	0 = closed	1	1	1	1	1	1	1	1	1	1	1	1	1	1	1
	Deliveries		6	10	6	8	2	7	8	4	9	13	11	12	6	12	6
		C-Section	1	4	0	1	1	2	3	1	2	3	6	3	5	2	1
PCM Hospital	Hospital status	0 = closed	1	1	1	1	1	1	1	1	1	1	1	1	1	1	1
	Deliveries		46	52	71	60	72	60	57	69	59	57	51	75	65	62	71
		C-Section	17	34	27	30	20	28	16	32	30	15	22	22	30	25	26

2014

16	17	18	19	20	21	22	23	24	25	26	27	28	29	30	31	32	33	34	35	36	37
1	1	1	1	1	1	1	1	1	1	1	1	1	1	1	1	1	1	1	1	1	1
12	3	8	7	7	9	16	12	2	11	10	6	7	12	4	1	3	1	12	3	7	7
1	2	2	3	3	0	0	0	0	0	0	0	2	2	0	2	0	0	0	1	1	0
1	1	1	1	1	1	1	1	1	1	1	1	1	1	1	1	1	1	1	1	1	1
32	28	38	31	42	32	39	40	24	26	29	21	20	31	11	25	22	23	36	33	32	15
0	0	0	0	0	8	9	7	6	0	1	1	0	0	0	0	0	2	2	1	2	0
1	1	1	1	1	1	1	1	1	1	1	1	1	1	1	1	1	1	1	1	1	1
3	5	4	3	3	0	4	1	4	5	1	1	6	5	4	1	3	7	2	1	3	7
0	3	2	1	0	2	0	2	0	3	0	1	0	0	0	1	0	0	1	0	0	2
1	1	1	1	1	1	1	1	1	1	1	1	1	1	1	1	1	1	1	1	1	1
12	7	9	2	4	5	1	7	9	6	11	14	4	6	5	2	9	11	4	7	10	3
7	5	3	6	6	5	2	6	11	3	6	4	0	2	4	3	7	8	1	0	4	1
1	1	1	1	1	1	1	1	1	1	1	1	1	1	1	1	1	1	1	1	1	1
1	4	3	5	5	1	5	4	1	2	2	1	0	0	0	0	1	0	2	1	2	0
0	0	0	0	0	0	0	0	0	0	0	0	0	0	0	0	0	0	0	0	0	0
1	1	1	1	1	1	1	1	1	1	1	1	1	1	1	1	1	1	1	1	1	1
4	7	3	7	5	9	6	6	3	7	4	6	6	9	5	3	10	6	9	8	3	5
2	6	2	5	6	1	3	3	1	0	5	3	0	1	3	2	1	3	2	2	5	3
1	1	1	1	1	1	1	1	1	1	1	1	1	1	1	1	1	1	1	1	1	0
5	1	4	4	2	6	2	1	2	4	1	3	5	4	5	2	1	1	2	0	1	0
3	3	3	2	2	2	2	3	4	3	3	3	0	0	0	2	1	1	2	0	0	0
1	1	1	1	1	1	1	1	1	1	1	1	1	1	1	1	1	1	1	1	1	1
27	28	31	38	46	41	44	42	35	38	30	37	34	27	40	35	34	28	32	30	34	40
7	4	6	12	9	11	10	7	5	10	2	3	7	12	10	18	10	12	8	2	10	10
1	1	1	1	1	1	1	1	1	1	1	1	1	1	1	1	1	1	1	1	1	1
7	25	9	18	32	11	12	13	12	14	10	10	17	5	11	4	8	7	12	10	7	6
4	3	2	6	7	8	0	0	5	0	2	0	0	4	6	1	0	3	3	3	0	2
1	1	1	1	1	1	1	1	1	1	1	1	1	1	1	1	1	1	1	1	1	1
12	20	17	15	15	18	25	19	21	18	14	19	9	19	16	17	12	18	16	12	15	11
5	0	4	2	0	2	1	2	0	1	0	0	0	1	0	6	2	0	3	2	0	0
1	1	1	1	1	1	1	1	1	1	1	1	1	1	1	1	1	1	1	1	1	1
5	2	0	2	3	3	0	1	1	2	0	0	0	0	1	0	2	2	2	0	1	2
0	2	0	1	1	1	0	0	0	0	0	0	0	0	1	0	2	1	2	0	1	2
1	1	1	1	1	1	1	1	1	1	1	1	1	1	1	1	1	1	1	1	1	1

1	0	1	7	6	8	7	13	20	18	13	11	14	9	4	1	0	7	0	4	3	1
2	0	1	0	1	1	1	0	5	1	2	0	0	0	0	1	2	0	0	1	1	0
1	1	1	1	1	1	1	1	1	1	1	1	1	1	1	1	1	1	1	1	1	1
10	18	14	12	7	11	14	7	4	15	0	7	12	4	11	10	7	10	6	0	1	10
3	2	4	3	0	1	3	1	3	3	0	0	3	1	1	2	0	0	0	0	3	4
1	1	1	1	1	1	1	1	1	1	1	1	1	1	1	1	1	1	1	1	0	0
5	6	7	9	7	3	8	10	7	8	10	13	7	7	6	3	7	3	4	3	0	0
7	7	1	11	13	7	8	13	7	8	9	0	5	5	3	3	5	12	5	4	0	0
1	1	1	1	1	1	1	1	1	1	1	1	1	1	1	1	1	1	1	1	1	1
1	3	3	2	4	3	5	3	1	2	1	3	3	3	1	2	1	2	1	0	1	0
1	1	2	1	2	1	1	2	0	1	1	2	2	2	0	1	1	0	1	0	1	0
1	1	1	1	1	1	1	1	1	1	1	1	1	1	1	1	1	1	1	1	1	1
15	13	22	13	7	16	11	18	9	9	11	10	4	10	8	5	10	8	6	6	1	2
6	7	5	7	10	7	11	13	4	4	6	5	3	4	6	4	6	4	9	2	1	3
1	1	1	1	1	1	1	1	1	1	1	1	1	1	1	1	0	0	0	0	0	0
7	2	3	5	4	3	1	9	1	6	2	5	4	0	4	2	0	0	0	0	0	0
0	0	1	1	0	0	0	2	1	0	0	2	0	0	1	0	0	0	0	0	0	0
1	1	1	1	1	1	1	1	1	1	1	1	1	1	1	1	1	1	1	1	1	1
19	25	29	18	11	14	10	25	9	10	11	16	15	21	18	12	15	20	25	14	9	3
0	0	0	7	2	5	4	7	2	4	1	2	14	2	1	3	1	2	1	0	0	0
1	1	1	1	1	1	1	1	1	1	1	1	1	1	1	1	1	1	1	1	1	1
17	29	16	32	21	29	15	25	25	22	15	13	17	19	21	14	11	0	0	0	0	0
10	18	8	18	16	14	8	22	15	13	10	5	7	15	8	2	4	0	0	0	0	0
1	1	1	1	1	1	1	1	1	1	1	1	1	1	1	1	1	1	1	1	1	1
6	5	6	7	3	8	1	5	12	4	4	7	7	5	7	9	8	11	8	14	12	1
0	1	0	5	3	0	2	0	8	0	4	0	1	0	0	2	0	2	0	6	0	3
1	1	1	1	1	1	1	1	1	1	1	1	1	1	1	1	1	1	1	1	1	1
3	4	4	4	5	1	4	1	4	2	4	4	2	3	3	3	1	1	1	0	0	0
0	1	1	0	0	0	0	0	0	0	0	0	0	0	0	0	0	0	0	0	0	0
1	1	1	1	1	1	1	1	1	1	1	1	1	1	1	1	1	1	1	1	1	1
2	4	4	5	5	6	4	5	10	2	6	3	4	3	6	2	3	6	3	3	1	1
0	0	0	0	1	1	0	1	5	1	0	0	2	0	0	0	0	0	0	0	0	0
1	1	1	1	1	1	1	1	1	1	1	1	1	1	1	1	1	1	1	1	1	1
4	3	5	9	8	6	4	9	9	4	2	9	10	7	5	3	6	8	2	2	1	0
1	1	1	2	2	2	1	2	2	0	2	2	3	2	1	1	0	1	0	0	0	0
1	1	1	1	1	1	1	1	1	1	1	1	1	1	1	1	1	1	1	1	1	1

2	11	6	17	11	7	3	6	9	8	7	8	17	11	7	3	6	9	8	7	8	5
3	3	5	2	1	1	1	7	2	2	0	4	1	1	2	5	1	3	1	5	5	2
1	1	1	1	1	1	1	1	1	1	1	1	1	1	1	1	1	1	1	1	1	1
25	30	35	29	28	35	43	37	35	26	27	26	25	19	30	31	29	24	16	10	17	18
8	5	7	6	6	4	8	8	8	12	9	3	7	3	2	6	2	5	3	2	4	4
1	1	1	1	1	1	1	1	1	1	1	1	1	1	1	1	1	1	1	1	1	1
13	15	13	17	12	16	11	14	10	11	10	9	11	11	10	8	8	10	0	0	0	0
4	6	3	3	2	3	3	1	2	8	3	4	1	1	1	2	4	2	1	1	3	2
1	1	1	1	1	1	1	1	1	1	1	1	1	1	1	1	1	1	1	1	1	1
5	6	11	12	2	8	8	12	3	14	7	3	9	6	5	1	3	2	3	2	4	3
0	0	0	2	1	1	1	3	2	1	1	0	1	0	0	0	0	0	0	0	0	0
0	0	0	0	0	0	0	0	1	1	1	1	1	1	1	1	1	1	1	1	1	1
0	0	0	0	0	0	0	0	3	2	3	4	7	1	4	2	1	2	6	2	3	0
0	0	0	0	0	0	0	0	1	1	4	2	2	7	1	0	3	0	3	0	2	5
1	1	1	1	1	1	1	1	1	1	1	1	1	1	1	1	1	0	0	0	0	0
13	13	12	9	13	9	13	12	11	6	6	9	4	10	5	0	2	0	0	0	0	0
0	0	0	0	0	0	0	1	0	0	0	0	1	0	0	0	0	0	0	0	0	0
1	1	1	1	1	1	1	1	1	1	1	1	1	1	1	1	1	1	1	1	1	1
23	20	15	32	21	19	27	30	12	14	15	9	13	21	17	9	11	24	10	32	10	8
0	0	0	0	0	0	0	0	0	0	0	0	0	0	0	0	0	0	0	0	0	0
1	1	1	1	1	1	1	1	1	1	1	1	1	1	1	1	1	1	1	1	1	1
14	5	11	13	4	12	8	13	14	10	5	6	8	11	7	17	7	5	7	6	8	4
6	2	4	3	2	2	2	7	3	2	0	0	5	3	1	7	3	0	3	3	6	1
1	1	1	1	1	1	1	1	1	1	1	1	1	1	1	1	1	1	1	1	1	1
64	79	86	72	82	103	99	89	72	74	74	72	77	72	72	68	74	82	70	55	55	65
23	33	22	36	23	27	41	46	32	40	27	34	32	31	38	21	20	27	27	20	40	26

38	39	40	41	42	43	44	45	46	47	48	49	50	51	52	1	2	3	4	5	6	7	8
1	1	1	1	1	1	1	1	1	1	1	1	1	1	1	1	1	1	1	1	1	1	1
5	6	9	8	8	11	9	4	2	3	4	3	3	2	2	1	1	2	2	10	12	7	13
1	0	3	1	3	2	0	1	2	1	1	2	1	0	1	0	2	1	2	2	3	0	3
1	1	1	1	1	1	1	1	1	1	1	1	1	1	1	1	1	1	1	1	1	1	1
29	36	33	41	19	49	52	18	38	42	38	36	41	38	24	12	30	24	20	18	32	28	26
2	2	4	3	4	9	4	8	6	5	8	6	8	10	8	7	8	11	7	6	4	6	7
1	1	1	1	1	1	1	1	1	1	1	1	1	1	1	1	1	1	1	1	1	1	1
12	4	6	5	2	4	6	8	12	10	8	9	6	6	7	4	6	4	5	6	4		6
3	2	1	3	3	2	1	1	2	2	1	2	1	0	0	0	0	1	2	3	3		1
1	1	1	1	1	1	1	1	1	1	1	1	1	1	1	1	1	1	1	1	1		
9	8	8	21	7	5	8	4	5	3	8	11		9	9		8	5	9	11	9		16
2	0	0	1	4	2	0	0	0	0	0	0	0	1	2		2	3	1	2	4		
1	1	1	1	1	1	1	1	1	1	1	1	1	1	1	1	1	1	1	1	1	1	1
2	0	0	1	0	1	2	4	0	2	0	0	0	3	0	0	0	0	0	0	0	0	0
0	0	0	0	0	0	0	0	0	0	0	0	0	0	0	0	0	0	0	0	0	0	0
1	1	1	1	1	1	1	1	1	1	1	1	1	1	1	1	1	1	1	1	1	1	1
9	8	6	4	8	9	7	10	13	8	11	20	18	11	13	8	6	2	4	6	4	7	3
5	8	3	2	3	5	3	2	4	9	5	2	1	3	12	7	6	3	4	4	3	3	3
0	0	1	1	1	1	1	1	1	1	1	1	1	1	1	1	1	1	1	1	1	1	1
0	0	0	0	0	0	0	4	3	2	2	5	5	5	2	10	12	10	11	10	10	10	10
0	0	0	0	0	0	0	3	5	4	3	4	4	4	2	3	5	6	5	5	5	5	5
1	1	1	1	1	1	1	1	1	1	1	1	1	1	1	1	1	1	1	1	1	1	1
50	64	38	30	0	39	38	38	34	19	17	22	16	18	1	0	4	4	3	1	5	4	1
12	16	13	9	0	14	19	16	13	9	11	1	0	3	0	0	0	0	0	0	2	1	0
1	1	1	1	1	0	0	0	0	1	0	0	1	1	1	1	1	1	1	1	1	1	1
5	11	3	7	10	0	0	0	0	3	4	3	0	0	0	1	5	3	2	0	2	0	3
2	1	4	3	0	0	0	0	0	1	0	3	0	0	0	0	0	1	1	0	0	1	0
1	1	1	1	1	1	1	1	1	1	1	1	1	1	1	1	1	1	1	1	1	1	1
14	14	13	10	15	9	10	22	18	13	14	12	13	13	19	14	18	12	11	10	20	10	16
0	0	0	0	0	3	1	0	1	2	1	2	0	2	0	0		4	2	2	1	0	1
1	1	1	1	1	1	1	1	1	1	1	1	1	1	1	1	1	1	1	1	1	1	1
3	2	1	2	1	5	3	2	4	9	8	7	7	9	3	3	7	6	8	16	58	61	10
1	2	2	4	3	0	0	4	2	4	4	5	2	4	2	3	2	2	3	0	0	0	1
1	1	1	1	1	1	1	1	1	1	1	1	1	1	1	1	1	1	1	1	1	1	1

2					2	1	2	3	2	3	3	0	4	1	0	0	4	4	3	4	4	4
1	1	1	0	0	0	0	0	0	0	0	0	0	0	0	0	0	0	0	0	0	0	0
1	1	1	1	1	1	1	1	1	1	1	1	1	1	1	1	1	1	1	1	1	1	1
4	2	6	4	7	0	0	0	0	1	4	0	0	0	6	0	6	2	2	2	0	4	3
1	3	2	3	1	0	0	0	0	0	0	0	0	0	1	0	2	2	2	1	0	1	1
0	0	0	0	0	0	0	0	0	0	0	1	1	1	1	1	1	1	1	1	1	1	1
0	0	0	0	0	0	0	0	0	0	0	3	6	5	4	8	6	7	9	10	12	6	5
0	0	0	0	0	0	0	0	0	0	0	0	0	0	0	0	0	0	0	0	0	0	0
0	0	0	0	0	0	0	0	0	0	1	1	1	1	1	1	1	1	1	1	1	1	1
0	0	0	0	0	0	0	0	0	0	1	2	0	0	4	2	1	2	2	3	1	2	1
0	0	0	0	0	0	0	0	0	0	0	0	0	0	2	1	0	1	1	1	1	0	0
1	1	1	1	1	1	1	1	1	1	1	1	1	1	1	1	1	1	1	1	1	1	1
8	4	16	13	3	0	0	0	6	4	8	18	6	7	27	13	25	23	8	15	7	9	11
6	2	11	13	2	0	0	0	0	3	2	1	1	0	8	2	4	8	8	4	4	5	8
0	0	0	0	0	0	0	0	0	0	0	0	0	0	0	0	0	0	0	0	0	0	0
0	0	0	0	0	0	0	0	0	0	0	0	0	0	0	0	0	0	0	0	0	0	0
0	0	0	0	0	0	0	0	0	0	0	0	0	0	0	0	0	0	0	0	0	0	0
1	1	1	1	1	1	1	1	1	1	1	1	1	1	1	1	1	1	1	1	1	1	1
15	26	14	20	4	8	7	4	6	7	6	8	8	5	10	7	18	19	8	8	10	18	12
5	4	2	3	8	3	3	2	4	3	2	3	2	0	2	3	4	9	4	4	6	3	9
1	1	1	1	0	0	0	0	0	0	0	0	0	0	0	0	0	0	0	0	0	0	0
0	0	0	0	0	0	0	0	0	0	0	0	0	0	0	0	0	0	0	0	0	0	0
0	0	0	0	0	0	0	0	0	0	0	0	0	0	0	0	0	0	0	0	0	0	0
1	1	1	1	1	1	1	1	1	1	1	1	1	1	1	1	1	1	1	1	1	1	1
8	2	5	3	2	0	4	7	8	7	8	10	3	6	3	7	3	3	4	11	9	6	5
5	1	6	2	2	0	3	3	2	2	0	0	2	0	0	3	0	0	1	3	0	1	0
1	1	1	1	1	1	1	1	1	1	1	1	1	1	1	1	1	1	1	1	1	1	1
1	0	2	1	2	3	2	2	1	2	4	7	2	3	4	6	3	4	3	5	2	3	
0	0	1	0	0	1	0	0	0	0	0	1	0	0	0	0	0	0	0	0	0	0	0
1	1	1	1	1	1	1	1	1	1	1	1	1	1	1	1	1	1	1	1	1	1	1
2	0	3	2	3	6	4	5	3	2	10	9	4	2	5	4	3	4	5	3	6	6	4
0	0	0	0	0	1	0	0	0	0	0	0	0	0	0	0	0	0	0	0	0	0	0
1	1	1	1	1	1	1	1	1	1	1	1	1	1	1	1	1	1	1	1	1	1	1
10	6	7	9	2	6	5	4	0	1	1	7	2	7	4	2	1	2	3	0	2	7	3
0	0	0	1	0	0	0	0	0	0	0	0	0	0	0	0	1	0	2	2	0	1	0
1	1	1	1	1	1	1	1	1	1	1	1	1	1	1	1	1	1	1	1	1	1	1

5	9	9	5	2	6	5	2	3	1	2	2	4	14	5	3	8	3	6	9	6	4	10
0	4	4	2	1	2	1	2	0	2	5	2	4	3	1	0	11	3	5	6	6	2	2
1	1	1	1	1	1	1	1	1	1	1	1				1	1	1	1	1	1	1	1
15	16	19	19	16	13	23	10	10	12	8	4				9	15	19	18	31	22	15	18
5	5	4	2	0	1	2	3	3	0	1	0				0	3	2	5	0	2	3	4
1	1	1	1	1	1	1	1	1	1	1	1	1	1	1	1	1	1	1	1	1	1	1
5	5	6	4	5	8	10	3	5	7	6	4	6	4	6	4	6	8	6	5	3	0	1
2	3	4	3	3	6	4	3	3	2	1	3	2	3	1	2	0	3	2	3	1	0	0
1	1	1	1	1	1	0	0	0	1	1	1	1	1	1	1	1	1	1	1	1	1	1
10	7	6	6	0	0	0	0	0	1	0	0	0	0	0		1	2	0	0	1	0	0
0	0	0	0	0	0	0	0	0	0	0	0	0	0	0		0	0	0	0	0	0	0
0	0	0	0	0	0	0	0	0	0	0	0	0	0	0	0	0	0	0	0	0	0	0
0	0	0	0	0	0	0	0	0	0	0	0	0	0	0	0	0	0	0	0	0	0	0
0	0	0	0	0	0	0	0	0	0	0	0	0	0	0	0	0	0	0	0	0	0	0
0	0	0	0	0	1	1	1	1	1	1	1	1	1	1	1	1	1	1	1	1	1	1
0	0	0	0	0	0	0	0	0	0	0	0	0	0	0	0	0	0	0	0	0	0	0
0	0	0	0	0	0	0	0	0	0	0	0	0	0	0	0	0	0	0	0	0	0	0
1	1	1	1	1	1	1	1	1	1	1	1	1	1	1	1	1	1	1	1	1	1	1
2	25	24	12	2	14	18	18	9	8	17	15	18	9	8	13	10	10	13	15	14	12	10
0	0	0	0	0	0	0	0	0	0	0	0	0	0	0	0	0	0	0	0	0		
1	1	1	1	1	1	1	1	1	1	1	1	1	1	1	1	1	1	1	1	1	1	1
5	9	7	12	11	11	8	5	8	11	12	15	14	18	28	18	11	12	10	7	14	10	5
1	2	3	5	4	2	3	1	1	5	6	8	8	5	11	4	5	8	5	3	9	4	2
1	1	1	1	1	1	1	1	1	1	1	1	1	1	1	1	1	1	1	1	1	1	1
69	84	65	87	102	68	70	66	75	68	61	62	55	62	62	88	70	60	62	38	49	57	56
21	21	38	28	22	24	36	31	28	18	18	23	20	16	62	23	25	25	37	15	23	26	28

2015

9	10	11	12	13	14	15	16	17	18	19	20
1	1	1	1	1	1	1	1	1	1	1	1
12	10	11	10	11	10	11	12	10	12	11	9
2	4	6	0	1	2	1	2	2	2	1	0
1	1	1	1	1	1	1	1	1	1	1	1
24	18	30	24	30	32	36	28	24	32	18	22
8	5	9	7	5	6	8	4	6	2	4	4
1	1	1	1	1	1	1	1	1	1	1	1
8	8	6	4	5	3	6	12	13	11		11
0	2	2	1	2	1	2	1	0	1		2
1	1	1	1				1	1	1	1	1
9	15	6	8					6	7	5	
2	3	4	2		0	3		3	2	4	
1	1	1	1	1	1	1	1	1	1	1	1
0	0	0	0	0	0	0	0	0	0	0	0
0	0	0	0	0	0	0	0	0	0	0	0
1	1	1	1	1	1	1	1	1	1	1	1
4	3	2	4	3	2	5	8	9	5	3	10
2	3	1	3	0	1	3	4	1	3	4	2
1	1	1	1	1	1	1	1	1	1	1	1
16	16	9	13	12	12	11	10	10	6	6	6
5	5	5	5	4	4	4	4	3	2	2	2
1	1	1	1	1	1	1	1	1	1	1	1
10	12	9	14	18	17	14	30	27	14	35	0
1	2	0	2	0	0	3	2	12	10	18	10
1	1	1	1	1	1	1	1	1	1	1	1
1	2	11	5	8	10	11	12	9	7	3	8
0	0	0	2	1	3	3	0	1	1	0	0
1	1	1	1	1	1	1	1	1			
13	11	11	4	8	10	11	12	9			
1	2	2	2	1	3	3	0	1			
1	1	1	1	1	1	1	1	1	1	1	1
16	39	31	51	11	55	46	18	24	64	54	43
3	3	2	2	2	1	5	2	2	5	2	2
1	1	1	1	1	1	1	1	1	1	1	1

5	0	2	0	1	3	2	6	2	7	7	7
0	0	0	0	0	0	0	1	0	0	2	0
1	1	1	1	1	1	1	1	1	1	1	1
1	1	4	0	1	3	5	4	4	4	7	10
1	1	0	0	0	0	3	1	1	0	2	0
1	1	1									
4	8	10									
0	0	2									
1	1	1	1	1	1	1	1	1	1	1	1
3	3	3	3	3	3	3	3	3	3	3	3
1	1	0	0	0	2	1	2	1	1	1	1
1	1	1	1	1	1	1	1	1	1	1	1
7	6	10	6	5	9	8	2	12	3	5	6
10	3	4	7	4	2	8	2	10	3	7	9
0	0	0	0	0	0	0	0	0	0	0	0
0	0	0	0	0	0	0	0	0	0	0	0
0	0	0	0	0	0	0	0	0	0	0	0
1	1	1	1	1	1	1	1	1	1	1	1
11	16	9	6	0	9	7	9	10	9	9	6
5	5	8	6	9	3	3	11	6	5	1	4
0	0										
0	0										
0	0										
1	1	1	1	1	1	1	1	1	1	1	1
9	12	4	8	11	6	6	9	16	17	14	14
1	0	5	1	0	5	3	3	2	2	4	3
1	1	1	1	1	1	1	1	1	1	1	1
3	2	3	4	6	7	8	8	8	7	7	8
0	0	0	0	0	0	0	0	0	0	0	0
1	1	1	1	1	1	1	1	1	1	1	1
5	9	8	8	8	3	4	4	4	3	4	2
0	2	1	1	2	0	0	2	0	0	0	1
1	1	1	1	1	1	1					
1	4	3	2	1	2	4					
0	2	2	0	1	0	2					
1	1	1	1	1	1	1	1	1	1	1	

6	13	11	3	5	7	7	16	15	19	17	
4	8	7	7	4	8	8	9	7	17	13	
1	1	1	1	1	1	1	1	1	1	1	1
19	14	21	16	23	29	21	30	36	44	42	35
2	4	3	3	9	5	1	4	8	13	10	7
1	1	1	1	1	1	1	1	1	1	1	1
3	3	4	4	3	3	2	2	2	2	1	3
1	0	1	1	0	1	1	0	1	1	1	1
1	0	1	1	1	1	1	1	1	1	1	1
0	0	0	1	2	1	0	1	2	0	1	1
0	0	0	0	0	0	0	0	1	0	0	1
0	0										
0	0										
0	0										
1	1	1	1	1	1	1	1	1	1	1	1
0	0	0	0	0	0	0	0	0	0	0	0
0	0	0	0	0	0	0	0	0	0	0	0
1	1	1	1	1	1	1	1	1	1	1	1
9	16	12	8	13	13	8	16	8	12	9	14
			0								
1	1	1	1	1	1	1	1	1	1	1	1
11	9	6	3	13	10	7	6	7	16	8	14
5	2	0	2	4	5	2	3	3	10	5	4
1	1	1	1	1	1	1	1	1	1	1	1
39	45	48	58	44	54	62	43	61	48	43	43
33	44	43	41	47	34	23	32	44	37	23	33
